# Development and Preliminary Validation of a MALDI-TOF MS Assay Using KTI as an Internal Standard for Serum M-Protein Light Chain Analysis in Multiple Myeloma: A Pilot Study

**DOI:** 10.3390/medicina62061057

**Published:** 2026-05-29

**Authors:** Jin Wang, Yiming Zhao, Shuanglian Xie, Huihui Liu, Mingyi Di, Bingjie Wang, Bo Tang, Weiwei Xie, Xiaoying Yang, Zhizhen Lai, Yujun Dong

**Affiliations:** Department of Hematology, Peking University First Hospital, Beijing 100032, China

**Keywords:** MALDI-TOF MS, multiple myeloma, M-protein, light chain, mass spectrometry, disease monitoring

## Abstract

*Background and Objectives*: Conventional assays for M-protein detection in multiple myeloma (MM), including serum immunofixation electrophoresis (sIFE) and serum free light-chain (sFLC) assays, have limitations in selected clinical settings. This pilot study aimed to develop and preliminarily validate a matrix-assisted laser desorption/ionization time-of-flight mass spectrometry (MALDI-TOF MS)-based workflow using Kunitz trypsin inhibitor (KTI) as an internal standard for patient-specific serum M-protein light-chain tracking, particularly in low-level post-treatment samples in which conventional assays may be negative or difficult to interpret. *Materials and Methods*: A total of 55 serum samples from 25 patients with MM were analyzed. Serum immunoglobulin light-chain species were enriched using mixed κ/λ affinity beads, followed by reduction, KTI-based calibration, and MALDI-TOF MS analysis. Quantitative performance was evaluated using purified IgG1 κ standards. Time-matched sIFE and sFLC ratio results were used for descriptive comparison. *Results*: After KTI-based calibration, patient-specific M-protein light-chain molecular masses could be consistently identified. The assay showed good linearity over the range of 0.20–10.00 μg/mL, with a calibration equation of y = 6.0228x + 0.1063 and an R^2^ of 0.9961. The limit of detection and limit of quantification were 0.002 μg/mL and 0.008 μg/mL, respectively. Intra-day and inter-day precision were acceptable, and recovery ranged from 96.0% to 101.2%. In selected low-level or discordant samples, including cases with therapeutic interference, polyclonal background, or non-secretory disease, MALDI-TOF MS provided exploratory complementary monitoring information. *Conclusions*: This KTI-calibrated MALDI-TOF MS workflow showed preliminary analytical performance within the validated low-concentration range and may serve as a complementary approach for patient-specific serum M-protein light-chain monitoring in selected clinical settings of MM. Larger independent studies are required before its clinical utility can be established.

## 1. Introduction

Multiple myeloma (MM) is the second most common hematological malignancy derived from plasma cells [1]. Its laboratory diagnostic features mainly include the presence of monoclonal immunoglobulin (M-protein) in serum or urine, together with infiltration of malignant plasma cells in the bone marrow [2]. Immunofixation electrophoresis (IFE) is a standard clinical method for the screening and monitoring of M-protein [3], and it can identify intact immunoglobulins as well as isolated heavy or light chains. Although IFE is effective for detecting most M-proteins, its sensitivity remains limited in certain settings because of immunoglobulin co-migration and the low serum concentrations that may occur with advances in chemotherapy and immunotherapy [4,5]. The serum free light-chain (sFLC) assay uses specific antibodies to quantify kappa (κ) and lambda (λ) free light chains and to calculate the κ/λ ratio, which is an important parameter for establishing clonality [6]. These antibodies recognize epitopes within the constant region of the light chain that are masked when bound to the heavy chain and exposed in the unbound state [7]. A substantial proportion of light-chain MM can be identified through an abnormal free light-chain ratio [8]. In addition, approximately 3% of patients with MM do not show detectable M-protein by IFE and are classified as non-secretory MM; however, these patients often present with abnormal sFLC ratios [9].

Over the past two decades, substantial progress has been made in mass spectrometry-based monitoring of M-protein [10,11]. The two principal analytical platforms are high-performance liquid chromatography-electrospray ionization-mass spectrometry (HPLC-ESI-MS) and matrix-assisted laser desorption ionization-time of flight-mass spectrometry (MALDI-TOF MS). One representative HPLC-ESI-MS approach is monoclonal immunoglobulin rapid accurate molecular mass (miRAMM), which improves target-protein resolution through liquid chromatographic separation [12]. In such approaches, both retention time and molecular mass can be used to define a monoclonal light chain [13]. Compared with MALDI-TOF MS, HPLC-ESI-MS is more susceptible to matrix effects, in which non-target compounds interfere with ionization and detection [14]. Such interference may arise from solvent effects, ion suppression or enhancement, and mass spectrometer-related factors [15,16,17]. In contrast, MALDI-TOF MS results are generally easier to analyze and interpret. In addition, MALDI-TOF MS requires only a short acquisition time per sample and has relatively lower instrument costs, making it suitable for large-scale clinical applications. Several clinical studies have already demonstrated the high sensitivity of MALDI-TOF MS for M-protein detection [18,19,20].

Immunoglobulins are composed of two heavy chains and two light chains, and each chain contains constant and variable regions. During B-cell development, the variable regions of both heavy and light chains are generated through specific immunoglobulin gene-segment rearrangements [21,22]. As a result, each clonal B cell possesses a unique rearrangement pattern and produces immunoglobulins with distinct and relatively stable molecular masses. This characteristic provides the basis for M-protein detection by mass spectrometry [23,24]. Given the patient-specific nature of M-protein, we designed a quantitative detection strategy using Kunitz trypsin inhibitor (KTI) as an internal standard for serum M-protein analysis. KTI is one of the best-characterized protease inhibitors derived from plant sources [25]. This 216-amino-acid protein has a molecular mass of approximately 20 kDa, which is clearly distinct from the typical molecular mass range of M-protein light chains (22–26 kDa) [26,27], thereby avoiding interference with M-protein signal recognition.

In this pilot study, KTI was incorporated as an internal standard into extracted immunoglobulin light chains (IMLCs), and the performance of MALDI-TOF MS was evaluated in 55 serum samples from patients with MM, with descriptive comparison against serum IFE and sFLC assays. We established a MALDI-TOF MS-based workflow for patient-specific M-protein light-chain molecular-mass tracking, with particular attention to low-level post-treatment samples in which sIFE may be negative or sFLC results may be difficult to interpret. This workflow showed preliminary analytical performance within a defined concentration range and may provide complementary information to conventional assays in selected clinical monitoring settings.

## 2. Materials and Methods

### 2.1. Samples

A total of 55 serum samples from 25 patients with multiple myeloma (MM) were analyzed by MALDI-TOF MS. Time-matched serum immunofixation electrophoresis (sIFE) and serum free light-chain ratio (sFLC ratio) results were available for 55 and 50 samples, respectively. This was an exploratory pilot study, and no independent validation cohort, predefined subgroup comparison, or statistical power calculation was included. Induction regimens were categorized as proteasome inhibitor-based (PI-based), proteasome inhibitor plus immunomodulatory drug-based (PI + IMiD-based), and daratumumab-based (Dara-based) therapies according to the main recorded treatment regimen. These categories should not be interpreted as mutually exclusive drug exposures, because daratumumab-based regimens could also include proteasome inhibitors and/or immunomodulatory drugs. The cohort included diverse M-protein types. Baseline clinical characteristics were collected and analyzed ( Supplementary Materials Table S1). Written informed consent was obtained from all participants. Clinical histories and serial laboratory results were retrieved from medical records. This study was approved by the Ethics Committee of Peking University First Hospital (No. 2021 (323)).

### 2.2. Enrichment and Reduction of Serum Immunoglobulin Light-Chain Species

**Figure 1:**
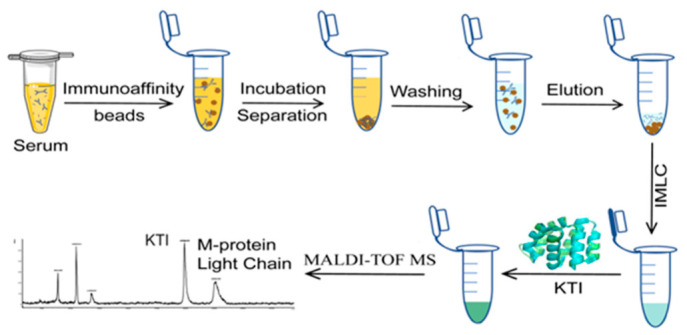
Workflow for enrichment of serum immunoglobulin light-chain species and MALDI-TOF MS analysis using KTI as an internal standard. Mixed immunoaffinity magnetic beads were used to enrich IMLC-related species from patient serum. The enriched light-chain species were obtained after washing, elution, and reduction, followed by the addition of KTI as an internal standard prior to MALDI-TOF MS analysis. IMLC, immunoglobulin light chain; KTI, Kunitz trypsin inhibitor. Different colors are used only to distinguish schematic components and procedural steps and do not represent quantitative variables.

An overview of the experimental workflow is presented in Figure 1. Briefly, 20 μL of patient peripheral blood serum was thoroughly mixed with 160 μL of 10 mM phosphate-buffered saline (PBS). The mixture was then incubated with CaptureSelect™ κ and λ light-chain affinity beads (Thermo Fisher Scientific, Waltham, MA, USA; Cat. Nos. 88843 and 88847), which had been pre-mixed at a 1:1 ratio, at a volume of 20 μL per sample [28]. According to the manufacturer’s specifications, the magnetic beads have a binding capacity of up to 10 μg of light chain per milligram of beads. After incubation for 45 min, the beads were washed three times with 200 μL of 10 mM PBS (pH 7.4) and then washed three additional times with HPLC-grade water to minimize nonspecific binding. The bound serum immunoglobulin light-chain species were subsequently eluted and reduced using 50 mM tris(2-carboxyethyl)phosphine (TCEP) prepared in 1% formic acid, and 30 μL of supernatant was collected as the light-chain solution.

In this workflow, mixed κ/λ affinity beads were used to enrich serum immunoglobulin light-chain species prior to reduction and MALDI-TOF MS analysis. This strategy was based on prior immunoenrichment-MS approaches for serum M-protein analysis, including nanobody-mediated MASS-FIX and mixed κ/λ bead enrichment, which have demonstrated that enriched immunoglobulin/light-chain fractions can reveal monoclonal molecular-mass patterns in samples from patients with monoclonal gammopathies while showing polyclonal peak distributions in control samples [19,20,28]. Accordingly, in the present assay, a light-chain signal was interpreted as an M-protein light-chain signal only when it met all of the following criteria: consistency with the patient’s known M-protein type, location within the expected immunoglobulin light-chain molecular-mass range, reproducible KTI-calibrated molecular mass, and longitudinal trackability from a baseline or clinically active sample. Thus, the assay was designed for patient-specific M-protein light-chain tracking rather than for indiscriminately assigning all bead-captured light chains as monoclonal or for analytically differentiating free light chains from heavy-chain-associated light chains. Pre-analytical and matrix-related factors, including protein binding, serum albumin concentration, temperature, and other sample-handling conditions, may affect enrichment or ionization efficiency; these factors were controlled by applying a standardized workflow but were not independently evaluated in this preliminary study.

### 2.3. Preparation of the Spiked Working Solution

KTI powder was dissolved in 1% formic acid to prepare a standard working solution at a concentration of 10 g/L. Then, 20 μL of this standard working solution was added to the eluate containing enriched serum immunoglobulin light-chain species and mixed thoroughly to obtain the spiked working solution.

### 2.4. MALDI-TOF MS Analysis

For MALDI-TOF MS analysis, 1 μL of the spiked working solution was mixed with 1 μL of α-cyano-4-hydroxycinnamic acid matrix at a concentration of 20 g/L. The mixture was spotted onto an MTP AnchorChip™ target and allowed to dry at room temperature before mass spectrometric analysis. Samples were analyzed using a MALDI-TOF mass spectrometer equipped with a SmartBeam-II laser system (Bruker Daltonics, Billerica, MA, USA) in positive-ion mode. The instrument parameters were set as follows: laser energy, 70%; laser shots, 2000 shots per spectrum; mass range, 15,000–30,000 *m*/*z*; full width at half maximum (FWHM), 1:10,000; and detector mode, linear positive-ion mode.

### 2.5. Quantitative Analysis and Data Processing

Data acquisition was performed using flexControl 3.0.0 software, and spectral processing was carried out using flexAnalysis 3.3 software (Bruker Daltonics, Bremen, Germany). The exact molecular masses of M-protein light chains were obtained by calibrating the spectra to the exact molecular mass of KTI (19,965.06 Da), which had been determined by high-resolution Q-Exactive MS. The signal intensities of the KTI peak (S1) and the IMLC peak (S2) were extracted using flexAnalysis 3.3 software.

For each patient, the patient-specific monoclonal light-chain peak was first identified from the baseline or clinically active sample according to the known M-protein type, the expected molecular-mass range of immunoglobulin light chains, and the reproducibility of the KTI-calibrated molecular mass. The same calibrated molecular-mass peak was then tracked in serial samples. Therefore, in post-treatment samples with reduced monoclonal burden or increased polyclonal background, the quantified signal corresponded to the predefined patient-specific monoclonal light-chain peak rather than the most abundant polyclonal peak.

To establish a quantitative MALDI-TOF assay targeting intact monoclonal light chains, calibration standards were prepared using IgG1 κ purified from the plasma of patients with MM (Sigma-Aldrich, St. Louis, MO, USA). Serial dilutions of the IgG1 κ stock were prepared as light-chain equivalents over a concentration range of 0.20–10.00 μg/mL, and KTI was added as an internal standard in desalted human serum. This concentration range was selected to evaluate low-level post-treatment monitoring scenarios rather than the full concentration spectrum of newly diagnosed or high-burden disease. This design was consistent with the intended application of low-level post-treatment or low-burden serum M-protein monitoring, in which conventional sIFE or sFLC-based interpretation may be negative, weak, or discordant. To minimize matrix interference, samples were desalted using 10,000 Da molecular weight cutoff centrifugal filters, mixed with α-cyano-4-hydroxycinnamic acid matrix at a 1:1 (*v*/*v*) ratio, and spotted in quintuplicate onto a stainless-steel target plate. MALDI-TOF spectra were acquired on a Bruker UltrafleXtreme™ instrument (Bruker Daltonics, Bremen, Germany) in linear positive-ion mode over a mass range of 15,000–30,000 Da, with 500 laser shots per spot. Quantitative analysis was based on the signal intensity ratio of the predefined patient-specific IMLC peak to the KTI peak. Values above the validated linear range were interpreted only as semi-quantitative estimates unless dilution-based reanalysis is performed.

### 2.6. Conventional Laboratory Assays: IFE and sFLC

All laboratory assays were performed according to the standard procedures of the Clinical Immunology Laboratory. Serum IFE results were interpreted by trained staff in the Department of Clinical Chemistry. Serum free light-chain (sFLC) quantification was performed by immunonephelometry using a BN™ ProSpec^®^ system (Siemens Healthineers, Erlangen, Germany) with Freelite^®^ reagents (The Binding Site Group Ltd., Birmingham, UK), and the results were interpreted in accordance with the relevant International Myeloma Working Group criteria. An elevated κ light-chain level together with an sFLC ratio > 1.56 was interpreted as being associated with κ-type M-protein, whereas an elevated λ light-chain level together with an sFLC ratio < 0.31 was interpreted as being associated with λ-type M-protein. In this study, the biological reference intervals for sFLC κ and λ were 6.7–22.4 mg/L and 8.3–27.0 mg/L, respectively.

## 3. Results

### 3.1. Overview of the Study Cohort and Mass Spectrometry Results

A total of 55 serum samples from 25 patients with multiple myeloma were analyzed by MALDI-TOF MS. The mass spectrometry results together with the corresponding clinical data are summarized in Supplementary Materials Table S2. Among samples with trackable patient-specific light-chain peaks, 15 were categorized as κ-type and 27 as λ-type according to the baseline M-protein type and the KTI-calibrated molecular-mass tracking strategy. The baseline clinical characteristics of the patient cohort are summarized in Table 1.

**Table 1:** Baseline clinical characteristics of the patient cohort.

Characteristic	Value
Age, years, median (range)	61 (56–65)
Age group, *n* (%)	
≤65 years	19 (76.0)
>65 years	6 (24.0)
Sex, *n* (%)	
Male	13 (52.0)
Female	12 (48.0)
Immunoglobulin type, *n* (%)	
IgG κ	2 (8.0)
IgG λ	5 (20.0)
IgA κ	1 (4.0)
IgA λ	5 (20.0)
LC-κ	5 (20.0)
LC-λ	5 (20.0)
IgD κ	1 (4.0)
Non-secretory	1 (4.0)
Revised International Staging System, *n* (%)	
I	2 (8.0)
II	8 (32.0)
III	13 (52.0)
Unknown	2 (8.0)
Induction treatment, *n* (%)	
PI-based	11 (44.0)
PI + IMiD-based	10 (40.0)
Daratumumab-based	4 (16.0)

Abbreviations: Ig, immunoglobulin; LC, light chain; PI, proteasome inhibitor; IMiD, immunomodulatory drug; κ, kappa; λ, lambda. Treatment categories were assigned according to the main recorded regimen and were not mutually exclusive exposures; daratumumab-based regimens could also include PI and/or IMiD agents.

### 3.2. KTI-Based Calibration Improved the Consistency of Molecular Mass Measurements

**Figure 2:**
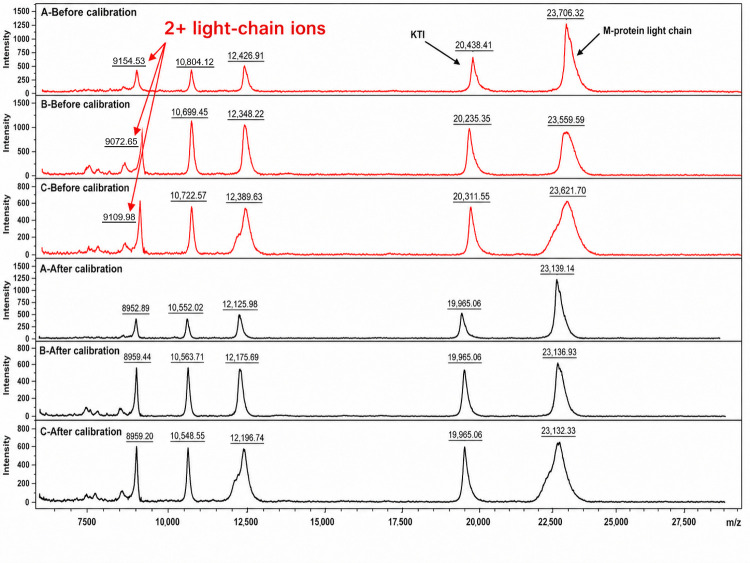
Representative MALDI-TOF MS spectra of the M-protein light chain in an IgG λ-type MM patient. (**A**) Baseline before treatment. (**B**,**C**) Post-treatment samples obtained after BCDx1 therapy and BCDx1 + RVDx2 therapy, respectively. Spectra before calibration are shown in red, and spectra after KTI-based calibration are shown in black. The sharp low-*m*/*z* peaks on the left side of the spectra mainly correspond to doubly charged light-chain ion species and were not used for quantitative calculation. Underlines indicate annotated peak *m/z* values. Quantitative analysis was based on the KTI-calibrated patient-specific singly charged IMLC peak. KTI, Kunitz trypsin inhibitor; IMLC, immunoglobulin light chain.

As shown in Figure 2, molecular mass variations of the M-protein light chain and KTI were evaluated in three serial samples from an IgG λ-type MM patient, including one baseline sample collected before treatment and two post-treatment samples obtained after BCDx1 therapy and BCDx1 + RVDx2 therapy. Before calibration with KTI, the measured molecular masses of the M-protein light chain were 23,706.32 Da, 23,559.59 Da, and 23,621.70 Da, while the corresponding KTI peaks were 20,438.41 Da, 20,235.35 Da, and 20,311.55 Da, respectively. After KTI-based calibration, the M-protein light chain peaks were stabilized at 23,139.14 Da, 23,136.93 Da, and 23,132.33 Da, respectively, whereas the KTI peak was consistently aligned at 19,965.06 Da.

After calibration using KTI as the internal standard, the corrected molecular masses of M-protein light chains were obtained for subsequent analysis (Supplementary Materials Table S3). The corrected molecular masses detected by MALDI-TOF MS were 19,965.06 Da for KTI, 23,378.45 ± 317.66 Da for κ-type M-protein light chains, and 22,856.44 ± 250.27 Da for λ-type M-protein light chains. The corresponding coefficients of variation were 1.36% for κ-type and 1.10% for λ-type M-protein light chains.

### 3.3. Quantitative Performance and Method Validation of the MALDI-TOF MS Assay

**Figure 3:**
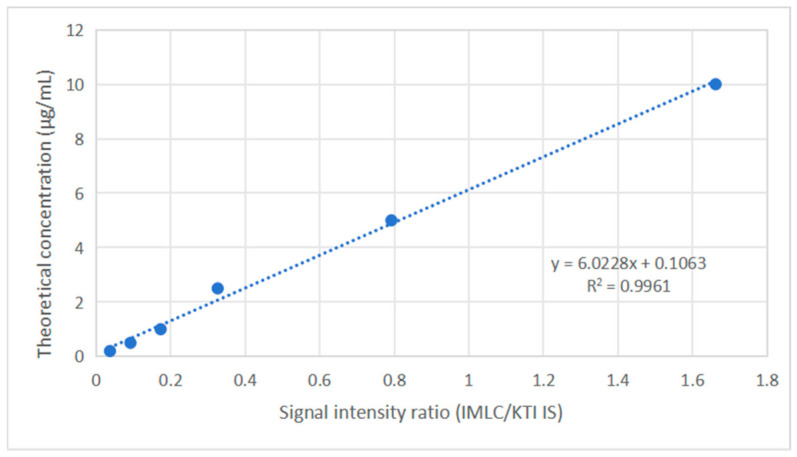
Calibration curve for quantitative analysis of immunoglobulin light chains by MALDI-TOF MS using KTI as an internal standard. The curve was generated using serial IgG1 concentrations. KTI, Kunitz trypsin inhibitor; IMLC, immunoglobulin light chain.

For quantitative assessment of M-protein light chains, the peak intensity ratio was used instead of peak area. The intensity ratio was normalized against the blank serum control and correlated with the theoretical concentrations for calibration curve construction. The calibration curve for quantitative detection of immunoglobulin light chains by MALDI-TOF MS using KTI as the internal standard is shown in Figure 3.

As summarized in Table 2, the assay showed a linear range of 0.20–10.00 μg/mL, with a calibration equation of y = 6.0228x + 0.1063 and a correlation coefficient (R^2^) of 0.9961. Based on 10 blank replicates, the limit of detection (LOD) and limit of quantification (LOQ) were calculated as 0.002 μg/mL and 0.008 μg/mL, respectively.

Method validation results are summarized in Table 3. At the tested concentration levels of 0.2, 5.0, and 10.0 μg/mL, the intra-day relative standard deviation (RSD) values ranged from 1.7% to 4.8%, and the inter-day RSD values ranged from 3.0% to 6.5%. Recovery rates ranged from 96.0% to 101.2%. Quantitative analysis of M-protein light chain concentrations in patient samples was subsequently performed using the established calibration equation.

The validated range of 0.20–10.00 μg/mL was intended for low-level monitoring after treatment. Patient-sample estimates exceeding 10.00 μg/mL were retained for descriptive purposes but were not interpreted as precise quantitative values, because dilution linearity and high-concentration nonlinearity were not evaluated in the present pilot study.

### 3.4. Exploratory Analysis of Selected Reference, Low-Level, or Discordant Samples

We next performed an exploratory descriptive analysis of selected samples used to define or track patient-specific MALDI-TOF MS peaks in clinically relevant low-level or discordant monitoring settings. Table 4 includes baseline or clinically active reference samples used to define the patient-specific molecular-mass peak, as well as post-treatment samples in which the same KTI-calibrated peak remained detectable while sIFE was negative or weakly positive, the κ/λ ratio was within the reference interval or discordant with the baseline M-protein type, or conventional interpretation was complicated by therapeutic antibody interference, non-secretory disease, renal dysfunction, or polyclonal background. These analyses were not intended to establish diagnostic sensitivity, specificity, or clinical superiority over conventional assays. The corresponding sIFE-based M-protein information, sFLC-κ concentration, sFLC-λ concentration, κ/λ ratio, KTI-calibrated IMLC molecular mass, and estimated IMLC concentration are summarized in Table 4.

Estimated IMLC concentrations were derived from the calibration equation. Values above 10.00 μg/mL exceeded the validated linear range and were interpreted only as semi-quantitative estimates. Quantitative serum M-protein concentrations by serum protein electrophoresis were not consistently available or measurable in these selected low-level samples; therefore, time-matched sIFE-based M-protein typing and sFLC results are provided as the main conventional reference information. For clarity, κ/λ ratio values within the laboratory reference interval are reported as within RI rather than as negative results.

## 4. Discussion

In this study, we developed and preliminarily validated a KTI-calibrated MALDI-TOF MS workflow for patient-specific serum M-protein light-chain tracking. The assay was developed as a complementary serum-based monitoring approach for low-burden post-treatment settings rather than as a replacement for established response assessment or as a method intended to cover the full concentration spectrum of newly diagnosed MM. In selected low-level or discordant monitoring scenarios, MALDI-TOF MS detected patient-specific light-chain peaks that were not fully captured by sIFE or sFLC ratio results. In patients P12 and P18, who received daratumumab-based therapy, MS-detectable patient-specific peaks were observed despite negative or less informative conventional results. In daratumumab-treated patients, therapeutic antibody interference may complicate electrophoretic interpretation, and previous work has shown that MALDI-TOF MS can distinguish daratumumab from endogenous M-proteins based on molecular mass [29]. In addition, patient-specific MS peaks were observed in samples with discordant conventional results, including the non-secretory case P21 and samples with renal dysfunction or polyclonal background. These observations suggest that the workflow may provide exploratory complementary information in selected challenging monitoring settings.

In patient P8, a detectable patient-specific MS peak was observed at three months after autologous stem cell transplantation despite negative conventional serum assays. However, the estimated concentration exceeded the validated linear range, and flow cytometry at the same time point was negative. Therefore, this observation should be interpreted only as an exploratory discordant finding, and its clinical significance remains uncertain. Longer follow-up and integrated assessment using established clinical, laboratory, and bone marrow-based methods are required before any disease-status interpretation can be made.

Although reproducible patient-specific molecular-mass peaks were used for longitudinal tracking, their detection should not be interpreted as direct evidence of active clonal disease in all clinical contexts. After treatment, immune reconstitution with re-emerging polyclonal immunoglobulins, renal function changes, and exposure to therapeutic monoclonal antibodies may alter the serum immunoglobulin background and complicate spectral interpretation. Thus, MS-detectable patient-specific peaks should be interpreted in relation to the baseline M-protein type, serial molecular-mass pattern, sIFE/sFLC results, treatment history, and overall clinical and marrow-based disease assessment.

The workflow established in this study has several methodological features. First, the study cohort included multiple M-protein isotypes, including non-secretory MM, intact immunoglobulins such as IgA, IgG, and IgD, and light-chain-only disease. Second, by combining immunoaffinity bead-based enrichment, disulfide-bond reduction, and MALDI-TOF MS detection, the method enables molecular-mass-based tracking of patient-specific light-chain peaks, consistent with prior LC-MS/MS and MALDI-TOF MS approaches for serum M-protein detection [30,31]. Third, KTI was incorporated as a practical internal reference for axis calibration and signal normalization within the same workflow. Nevertheless, because KTI is not an isotopically labeled or structurally identical internal standard, the present workflow should be considered a preliminary analytical approach for relative/semi-quantitative monitoring rather than a fully validated absolute quantification method.

The present serum MALDI-TOF MS workflow should also be distinguished from guideline-defined minimal residual disease (MRD) assessment. Current MRD methods in MM, including next-generation flow cytometry (NGF) and next-generation sequencing (NGS), evaluate residual clonal plasma cells in bone marrow at standardized sensitivity thresholds [32,33]. Our approach provides serum-based tracking of patient-specific M-protein light-chain molecular-mass signals and should not be considered a replacement for NGF/NGS-based MRD testing. The relationship between this serum MS signal and standardized MRD status requires prospective validation.

Several limitations of this study should be acknowledged. First, this was a single-center pilot study including 55 serum samples from 25 patients, without an independent validation cohort, predefined subgroup analysis, or statistical power calculation. Therefore, the present data are insufficient to establish diagnostic sensitivity, specificity, or clinical superiority over conventional assays. Second, the MALDI-TOF MS-based IMLC signal and sFLC results are not analytically equivalent: sFLC assays quantify unbound κ and λ free light chains, whereas the present workflow detects bead-enriched serum immunoglobulin light-chain species after reduction and tracks a patient-specific molecular-mass peak. Therefore, formal quantitative agreement analyses such as Bland–Altman or Passing–Bablok regression were not performed in this preliminary dataset. Third, although the method showed good linearity, precision, and recovery within 0.20–10.00 μg/mL, values above this range were interpreted only as semi-quantitative estimates unless dilution-based reanalysis is performed; dilution linearity and high-concentration nonlinearity were not systematically evaluated. Fourth, KTI is a non-isotopic internal standard and may not fully correct for matrix effects, ionization variability, or enrichment-related bias. Fifth, urine samples, renal function, treatment effects such as daratumumab exposure, serum albumin concentration, polyclonal immunoglobulin background, inter-instrument reproducibility, between-run calibration, and QC-based batch performance were not systematically evaluated. Finally, the follow-up period was limited and OS, PFS, and other clinical outcomes were not analyzed. Larger prospective studies with independent validation cohorts, standardized QC procedures, dilution-linearity testing, and longitudinal outcome assessment are required. The KTI-related technologies have been disclosed as patented technologies; this patent status does not affect the reported experimental observations, but independent external validation will be important to further evaluate the robustness and generalizability of the workflow.

## 5. Conclusions

This pilot study developed and preliminarily validated a KTI-calibrated MALDI-TOF MS workflow for patient-specific serum M-protein light-chain tracking in multiple myeloma. The workflow enabled molecular-mass-based monitoring and showed acceptable preliminary analytical performance within the validated low-concentration range. In selected low-level post-treatment or discordant samples, it provided exploratory complementary information when conventional assays were negative, weakly positive, discordant, or potentially affected by therapeutic interference. These findings support further evaluation of this approach, but larger independent cohorts, standardized quality-control procedures, dilution-linearity validation, and longer clinical follow-up are required before its clinical utility and its relationship with guideline-defined MRD assessment can be established.

## 6. Patents

Technologies related to the use of KTI are protected under Chinese patents 2024106536694, 2024106533836, and 2024106529101.

## Figures and Tables

**Table 2:** Analytical performance of the MALDI-TOF MS assay.

Parameter	Value/Range
Linear range	0.20–10.00 μg/mL
Calibration equation	y = 6.0228x + 0.1063
Correlation coefficient (R^2^)	0.9961
SD_blank	0.005
Limit of Detection (LOD)	0.002 μg/mL (S/N ≥ 3)
Limit of Quantification (LOQ)	0.008 μg/mL (S/N ≥ 10)

Abbreviations: LOD, limit of detection; LOQ, limit of quantification; S/N, signal-to-noise ratio.

**Table 3:** Precision and recovery of the MALDI-TOF MS assay.

Nominal Concentration (μg/mL)	Analysis Type	Measured Concentration (μg/mL)	RSD (%)	Recovery (%)
0.2	Intra-day	0.19, 0.21, 0.20	4.8	98.5
	Inter-day	0.18, 0.22, 0.19	6.5	96.0
5.0	Intra-day	4.85, 5.10, 4.95	2.2	99.3
	Inter-day	4.90, 5.25, 4.80	3.8	101.2
10.0	Intra-day	10.10, 9.85, 10.20	1.7	100.2
	Inter-day	9.95, 10.40, 9.70	3.3	99.7

**Table 4:** Exploratory analysis of selected reference, low-level, or discordant samples with detectable patient-specific MALDI-TOF MS peaks.

Patient	Sample ID	Time Point	Conventional Serum Findings	IMLC (Da)	IMLC (μg/mL)	Interpretation
P3	5	BCDx2	sIFE: LC-κ; sFLC-κ: 439 mg/L; sFLC-λ: 171 mg/L; κ/λ ratio: 2.57	23,267.48	2.08	Conventional assays positive; patient-specific κ-type MS peak detectable for longitudinal tracking
P3	6	BCDx4	sIFE: negative; sFLC-κ: 78.5 mg/L; sFLC-λ: 22.6 mg/L; κ/λ ratio: 3.47	23,267.48	1.81	sIFE-negative sample with κ-biased sFLC ratio; patient-specific MS peak detectable
P6	11	Biochemical recurrence	sIFE: LC-κ; sFLC-κ: 522.5 mg/L; sFLC-λ: 10.7 mg/L; κ/λ ratio: 48.83	23,248.63	0.96	Clinical recurrence/reference sample; patient-specific κ-type MS peak detectable
P6	12	2xRVD	sIFE: negative; sFLC-κ: /mg/L; sFLC-λ: /mg/L; κ/λ ratio: /	23,249.63	1.84	sIFE-negative sample without time-matched sFLC data; patient-specific MS peak detectable
P6	13	Before ASCT	sIFE: negative; sFLC-κ: 292.5 mg/L; sFLC-λ: 8.91 mg/L; κ/λ ratio: 32.83	23,250.63	1.34	sIFE-negative sample with κ-biased sFLC ratio; patient-specific MS peak detectable
P2	3	Baseline	sIFE: IgAλ; sFLC-κ: 6.56 mg/L; sFLC-λ: 30.6 mg/L; κ/λ ratio: 0.21	22,639.69	2.23	Reference sample for defining patient-specific λ-type MS peak
P2	4	RVDx2	sIFE: IgAλ; sFLC-κ: 7.26 mg/L; sFLC-λ: 13.2 mg/L; κ/λ ratio: within RI	22,639.69	1.01	sIFE-positive low-level sample with κ/λ ratio within RI; patient-specific MS peak detectable
P16	34	Baseline	sIFE: IgGλ; sFLC-κ: 9 mg/L; sFLC-λ: 118 mg/L; κ/λ ratio: 0.08	23,153.39	13.33 †	Reference sample for defining patient-specific λ-type MS peak; semi-quantitative estimate
P16	35	BCDx1	sIFE: IgGλ; sFLC-κ: 8.23 mg/L; sFLC-λ: 23.3 mg/L; κ/λ ratio: within RI	23,153.39	5.72	sIFE-positive sample with κ/λ ratio within RI; patient-specific MS peak detectable
P16	36	BCDx1 + RVDx2	sIFE: IgGλ; sFLC-κ: 18.3 mg/L; sFLC-λ: 19 mg/L; κ/λ ratio: within RI	23,153.39	7.07	sIFE-positive sample with κ/λ ratio within RI; patient-specific MS peak detectable
P22	48	Baseline	sIFE: IgGλ + LC-λ; sFLC-κ: 6.37 mg/L; sFLC-λ: 49.3 mg/L; κ/λ ratio: 0.13	22,823.86	4.47	Reference sample for defining patient-specific λ-type MS peak
P22	49	BCDx1	sIFE: IgGλ; sFLC-κ: 18.8 mg/L; sFLC-λ: 34.8 mg/L; κ/λ ratio: within RI	22,823.86	3.09	sIFE-positive sample with κ/λ ratio within RI; patient-specific MS peak detectable
P4	7	Baseline	sIFE: IgGλ + LC-λ; sFLC-κ: 9.73 mg/L; sFLC-λ: 3050 mg/L; κ/λ ratio: 0.0032	22,249.51	6.79	Reference sample for defining patient-specific λ-type MS peak
P4	8	Ddx8	sIFE: negative; sFLC-κ: 4.92 mg/L; sFLC-λ: 7.1 mg/L; κ/λ ratio: within RI	22,249.51	0.47	sIFE-negative sample with κ/λ ratio within RI; patient-specific MS peak detectable
P8	16	Before ASCT	sIFE: IgAλ; sFLC-κ: 7.75 mg/L; sFLC-λ: 17.3 mg/L; κ/λ ratio: within RI	22,569.98	3.23	Reference sample for defining patient-specific λ-type MS peak; κ/λ ratio within RI
P8	17	After ASCT 3 months	sIFE: negative; sFLC-κ: 11.6 mg/L; sFLC-λ: 15.4 mg/L; κ/λ ratio: within RI	22,569.98	10.63 †	Exploratory discordant finding; clinical significance uncertain
P10	21	Baseline	sIFE: LC-κ; sFLC-κ: >4575.0 mg/L; sFLC-λ: 22.8 mg/L; κ/λ ratio: >200.658	23,278.21	5.59	Reference sample for defining patient-specific κ-type MS peak
P10	22	BCDx4	sIFE: negative; sFLC-κ: 22.2 mg/L; sFLC-λ: 16.1 mg/L; κ/λ ratio: within RI	23,278.21	2.47	sIFE-negative sample with κ/λ ratio within RI; patient-specific MS peak detectable
P10	23	Before ASCT	sIFE: negative; sFLC-κ: 26.2 mg/L; sFLC-λ: 32 mg/L; κ/λ ratio: within RI	23,278.21	1.89	sIFE-negative sample with κ/λ ratio within RI; patient-specific MS peak detectable
P21	46	RVCD*2	sIFE: negative; sFLC-κ: /mg/L; sFLC-λ: /mg/L; κ/λ ratio: /	23,284.53	2.09	Detectable serum MS peak in non-secretory MM; no time-matched sFLC data
P21	47	Baseline	sIFE: negative; sFLC-κ: 6.31 mg/L; sFLC-λ: 8.02 mg/L; κ/λ ratio: within RI	23,284.53	1.61	Detectable serum MS peak in non-secretory MM; κ/λ ratio within RI
P24	52	Baseline	sIFE: LC-λ; sFLC-κ: 6.9 mg/L; sFLC-λ: 5610 mg/L; κ/λ ratio: 0.0012	22,660.59	17.01 †	Reference sample for defining patient-specific λ-type MS peak; semi-quantitative estimate
P24	53	BDx1, DRVDx2 before ASCT	sIFE: negative; sFLC-κ: 9.39 mg/L; sFLC-λ: 26.4 mg/L; κ/λ ratio: within RI	22,660.59	1.34	sIFE-negative sample with κ/λ ratio within RI; patient-specific MS peak detectable
P12	26	Baseline	sIFE: LC-λ; sFLC-κ: 21.8 mg/L; sFLC-λ: 2060 mg/L; κ/λ ratio: 0.01	22,900.8	4.95	Reference sample before daratumumab-based therapy; patient-specific λ-type MS peak detectable
P12	27	DRDx2	sIFE: IgGκ; sFLC-κ: /mg/L; sFLC-λ: /mg/L; κ/λ ratio: /	22,900.8	1.71	Therapeutic antibody interference suspected; patient-specific λ-type MS peak detectable
P12	28	/	sIFE: negative; sFLC-κ: 14.5 mg/L; sFLC-λ: 40.2 mg/L; κ/λ ratio: within RI	22,900.8	1.55	Post-treatment sample with negative sIFE; patient-specific λ-type MS peak detectable
P18	39	/	sIFE: LC-κ; sFLC-κ: 352 mg/L; sFLC-λ: 37.2 mg/L; κ/λ ratio: 9.46	23,287.05	0.54	Reference sample before daratumumab-based therapy; patient-specific κ-type MS peak detectable
P18	40	DVDx2	sIFE: IgGκ; sFLC-κ: 25.1 mg/L; sFLC-λ: 26.1 mg/L; κ/λ ratio: within RI	23,287.05	0.56	Therapeutic antibody interference suspected; patient-specific κ-type MS peak detectable
P7	14	Baseline	sIFE: IgAλ; sFLC-κ: 81.1 mg/L; sFLC-λ: 262.5 mg/L; κ/λ ratio: 0.31	23,038.01	1.53	Reference sample with polyclonal background; patient-specific λ-type MS peak detectable
P7	15	BDx3 + BCDx1	sIFE: IgAλ; sFLC-κ: 85 mg/L; sFLC-λ: 77.4 mg/L; κ/λ ratio: within RI	23,038.01	1.75	sIFE-positive sample with κ/λ ratio within RI under polyclonal background; patient-specific MS peak detectable
P11	24	Baseline	sIFE: LC-κ; sFLC-κ: 797 mg/L; sFLC-λ: 114 mg/L; κ/λ ratio: 6.99	23,306.97	0.75	Reference sample with polyclonal background; patient-specific κ-type MS peak detectable
P11	25	BCDx6 + DVDx1	sIFE: negative; sFLC-κ: 42.9 mg/L; sFLC-λ: 62.7 mg/L; κ/λ ratio: within RI	23,306.97	1.71	sIFE-negative sample with κ/λ ratio within RI under polyclonal background; patient-specific MS peak detectable
P15	32	Baseline	sIFE: LC-λ; sFLC-κ: 20.22 mg/L; sFLC-λ: 9876.6 mg/L; κ/λ ratio: 0.002	22,967.37	26.13 †	Reference sample with polyclonal background; patient-specific λ-type MS peak detectable; semi-quantitative estimate
P15	33	BCDx2	sIFE: negative; sFLC-κ: 34.1 mg/L; sFLC-λ: 35.9 mg/L; κ/λ ratio: within RI	22,967.37	3.34	sIFE-negative sample with κ/λ ratio within RI under polyclonal background; patient-specific MS peak detectable

IMLC concentrations were estimated using the calibration equation. † Values above 10.00 μg/mL exceeded the validated linear range and were interpreted as semi-quantitative estimates only. Quantitative serum M-protein levels by SPEP were not consistently available or measurable in these selected low-level samples. Abbreviations: sIFE, serum immunofixation electrophoresis; sFLC, serum free light chain; IMLC, immunoglobulin light chain; ASCT, autologous stem cell transplantation; RI, reference interval; SPEP, serum protein electrophoresis; MM, multiple myeloma; BCD, bortezomib, cyclophosphamide, and dexamethasone; BD, bortezomib and dexamethasone; RVD, lenalidomide, bortezomib, and dexamethasone; RVCD, lenalidomide, bortezomib, cyclophosphamide, and dexamethasone; DVD, daratumumab, bortezomib, and dexamethasone; DRD, daratumumab, lenalidomide, and dexamethasone; DRVD, daratumumab, lenalidomide, bortezomib, and dexamethasone.

## Data Availability

Data will be made available on reasonable request.

## Supplementary Materials

The following supporting information can be downloaded at: https://www.mdpi.com/article/10.3390/medicina62061057/s1, Table S1: Baseline clinical characteristics of the patients with multiple myeloma included in this study; Table S2: Mass spectrometry results and corresponding serum immunofixation and serum free light-chain findings for all analyzed samples; Table S3: KTI-calibrated M-protein light-chain molecular masses and related clinical findings in selected evaluable samples.
